# USSC-YOLO: Enhanced Multi-Scale Road Crack Object Detection Algorithm for UAV Image

**DOI:** 10.3390/s24175586

**Published:** 2024-08-28

**Authors:** Yanxiang Zhang, Yao Lu, Zijian Huo, Jiale Li, Yurong Sun, Hao Huang

**Affiliations:** 1 College of Civil Engineering, Central South University of Forestry & Technology, Changsha 410004, China; yxzhang1017@163.com; 2 College of Information Science and Technology, Shihezi University, Shihezi 832003, China; shzu_luyao@163.com; 3 College of Water Conservancy and Transportation, Zhengzhou University, Zhengzhou 450001, China; huo123@stu.zzu.edu.cn (Z.H.); 17639776435@163.com (J.L.); 4 College of Computer and Mathematics, Central South University of Forestry & Technology, Changsha 410004, China

**Keywords:** deep learning, machine vision, YOLOv5s, multi-scale, intelligent management

## Abstract

Road crack detection is of paramount importance for ensuring vehicular traffic safety, and implementing traditional detection methods for cracks inevitably impedes the optimal functioning of traffic. In light of the above, we propose a USSC-YOLO-based target detection algorithm for unmanned aerial vehicle (UAV) road cracks based on machine vision. The algorithm aims to achieve the high-precision detection of road cracks at all scale levels. Compared with the original YOLOv5s, the main improvements to USSC-YOLO are the ShuffleNet V2 block, the coordinate attention (CA) mechanism, and the Swin Transformer. First, to address the problem of large network computational spending, we replace the backbone network of YOLOv5s with ShuffleNet V2 blocks, reducing computational overhead significantly. Next, to reduce the problems caused by the complex background interference, we introduce the CA attention mechanism into the backbone network, which reduces the missed and false detection rate. Finally, we integrate the Swin Transformer block at the end of the neck to enhance the detection accuracy for small target cracks. Experimental results on our self-constructed UAV near–far scene road crack i(UNFSRCI) dataset demonstrate that our model reduces the giga floating-point operations per second (GFLOPs) compared to YOLOv5s while achieving a 6.3% increase in mAP@50 and a 12% improvement in mAP@ [50:95]. This indicates that the model remains lightweight meanwhile providing excellent detection performance. In future work, we will assess road safety conditions based on these detection results to prioritize maintenance sequences for crack targets and facilitate further intelligent management.

## 1. Introduction

Road infrastructure is an indispensable foundation for economic development, and countries invest enormous resources in road construction projects every year [1]. However, the increase in traffic volume and the prolongation of road infrastructure usage have led to an alarming rise in the prevalence of road cracks [2,3]. Cracks can be classified into five distinct categories: alligator crack, longitudinal crack, transverse crack, pothole, and patching [4]. Each type of crack has different detection needs and treatment priorities. The first three types of cracks are the main targets in the field of road crack detection, in which longitudinal and transverse cracks that are linear in shape are often a sign of structural damage to the road, and timely treatment before the crack reaches a critical size that may endanger road safety or significantly increase the cost of maintenance not only reduces the occurrence of safety accidents but also greatly reduces the cost of maintenance of the road, so linear cracks are the main area of research in the field of image vision [5,6]. If this linear crack is not controlled in a timely and effective manner, it will not only weaken the bearing capacity of the road but also lead to disruption in the road surface, thereby creating serious safety hazards [7], and may eventually lead to the road being scrapped [8]. Therefore, in civil engineering practice, the identification of linear cracks is a key initiative to ensure the safety of road structures and extend their service life [9].

Traditional road crack detection is mainly based on the inspector’s visual observation; although the accuracy rate is high, the efficiency is low and it needs to consume great human and material resources [10]. As the number of roads continues to grow, it has become evident that the current method for efficient detection is insufficient to meet the rising demand [11]. Consequently, a new approach based on digital image processing has emerged.

Traditional digital image processing methods, such as threshold segmentation, edge detection, and morphological operations, have been effective in the field of road crack detection [12]. Kusumaningrum et al. [13] proposed a road crack detection model based on the degree of category damage by converting RGB images captured by a digital camera into grayscale images, normalizing the images and determining the damage classification, and, finally, classifying the damage based on the grayscale thresholds using the K-NN algorithm, which achieves a significant improvement in accuracy. Liu et al. [14] addressed the issue of the inadequate performance of automatic crack detection by extracting crack information at multiple scales through thresholding at varying scales and employing the concept of edge detection to integrate crack characteristics at different scales. Zhong et al. [15] put forth an algorithm that can accurately detect cracks in various orientations based on mobile laser scanning (MLS) data. This is achieved by assigning two-dimensional indices in accordance with the angle of laser scanning or acquisition time and extracting crack features through the integration of morphological filtering, sparse algorithms, and Freeman coding. Mohamed et al. [16] used Beamlet algorithms to improve the grayscale representation of road cracks and to reduce the noise in the image, combined with a dedicated crack segmentation network to classify the pavement cracks, which was effective in detecting different types of cracks. Although traditional image processing methods have achieved satisfactory results in the field of road crack detection, their generalization ability and robustness are relatively weak due to their complexity and dependence on specific conditions [17]. Therefore, to improve the performance of crack detection, researchers have turned their attention to deep-learning algorithms.

Deep-learning algorithm [18] can autonomously discern and efficiently extract image features and has made significant advancements in the domain of image processing, particularly in object detection. Consequently, the deployment of this algorithmic approach to crack detection in intricate backgrounds is anticipated to demonstrate enhanced resilience and precision. Such an implementation can not only adapt to complex and evolving image contexts but also enhance the efficiency and dependability of crack detection.

Single-stage deep-learning algorithms represent a significant subcategory of deep-learning algorithms. Among these, algorithms such as RetinaNet [19], YOLO series [20,21], EfficientDet [22], and SSD [23] have demonstrated exceptional performance in real-time processing scenarios due to their high-speed processing capabilities. This has attracted numerous researchers to pursue further exploration and innovation, thereby advancing the continuous progress and development of deep-learning single-stage algorithms. Zhu et al. [24] put forth a novel Crack U-Net model that exhibits remarkable precision in crack detection. The basic convolution module of the network is composed of a module based on the U-Net network, residual blocks, and mini-U. The dilated convolution is used to replace the traditional convolution to fully capture the edge information of the crack. The resulting network achieves excellent accuracy on the CRACK500 and CFD datasets. Fan et al. [25] conducted an evaluation and comparison of the performance of 30 state-of-the-art (SoTA) DCNNs for road crack detection. The results demonstrate that all evaluated DCNNs exhibit comparable performance with a limited amount of training data, with PNASNet achieving the optimal balance between speed and accuracy. Li et al. [26] developed a novel road crack detection model, DenxiDeepCrack, based on data collected using UAV technology. The model demonstrated effective performance in detecting road cracks on both the CrackTree260 dataset and a self-built dataset. Hammouch et al. [27] put forth a methodology for the automated identification and categorization of flexible pavement fissures in Morocco, employing a CNN and pre-training the VGG-19 network through transfer learning. This approach yielded remarkable detection outcomes on the self-constructed dataset. Lv et al. [28] proposed a mask-region-based convolutional neural network (Mask R-CNN) model based on the mask region, to classify the types of road crack damage and elucidate the design concept of deep-learning technology in road crack detection tasks. The model demonstrated high detection accuracy for different types of road cracks. Li et al. [29] proposed a novel algorithm for the precise detection of road cracks. The proposed algorithm is based on the gm-resnet network and introduces a global attention mechanism to facilitate the extraction of high-latitude features across channels and spaces. Additionally, the algorithm employs a focus loss function to address the issue of category imbalance, resulting in high accuracy on the Concrete Structure Spalling and Cracking (CSSC) dataset. Chen et al. [30] devised a novel neural network for pixel-level pavement crack detection, which ingeniously fuses the strengths of the codec network architecture and attention mechanism to more accurately and efficiently extract crack pixels. Experimental outcomes demonstrate that the network exhibits remarkable detection capabilities. The research conducted by these scholars has yielded favorable detection outcomes on public datasets and self-created datasets, providing robust technical support for road maintenance and management. This indicates that the field of road crack detection is progressing towards a more intelligent and automated future.

Accurately identifying tiny cracks poses a big challenge in road crack detection. Often overlooked for their small size, these cracks signal road deterioration. Early detection is crucial to prevent major damage and ensure road safety [31]. Therefore, the detection of small cracks has become the focus of current research. Zhang et al. [32] put forth the notion that the CTCD-Net, with its innovative attention mechanism and cross-layer fusion, can precisely capture microcrack features while effectively reducing background noise, significantly improving detection accuracy and completeness. Ciocarlan et al. [33] developed an add-on NFA model for the detection of small targets. The model introduces an *a contrario* decision criterion, which enhances the sensitivity of feature mapping, minimizes false positives, and markedly improves the accuracy of small target detection. Li et al. [34] designed a deep-learning model based on CrackTinyNet (CrTNet). The model incorporates the BiFormer converter with an optimized loss function and the Space-to-Depth Conv technology, demonstrating distinctive capabilities in microcrack detection. He et al. [35] put forth a novel UAV-based MUENet algorithm. The algorithm has markedly enhanced the precision, velocity, and adaptability of road crack detection through its main and auxiliary dual-path module (MADPM), non-uniform fusion structure, and efficient E-SimOTA strategy. It has established a new standard for the identification of minute target cracks on the pavement. These investigations into the detection of small targets have not only yielded noteworthy outcomes, but, moreover, have provided a substantial impetus for the advancement of real-time and precise road crack detection technology.

While progress has been made in the field of road crack detection, the current automated identification methods still have some limitations due to the complexity and variability of the shapes, sizes, and backgrounds of pavement cracks. Especially in large-scale application scenarios that require a fast response and real-time processing, traditional models tend to have high computational complexity and high resource consumption, making it difficult to be lightweight while ensuring high accuracy; models that can be lightweight also tend to exhibit problems such as insufficient robustness and a high false detection rate. Therefore, the development of a model that can accurately detect line cracks of different scales under complex road surface backgrounds and meet the demand for being lightweight has become an urgent pain point in the field of road crack detection [36]. To address this issue, this paper puts forth a novel lightweight road crack detection model, USSC-YOLO, which is based on the YOLOv5s model and UAV aerial road crack data. This model is capable of the real-time and rapid monitoring of road cracks at different scales in complex backgrounds while ensuring optimal processing speed, especially for linear cracks. The contributions of this study are summarized as follows:

1. To detect cracks at varying scales and expand the crack detection range of a single image, we employed the use of drones to capture images of road cracks from both close and distant perspectives. Subsequently, we constructed a UNFSRCI dataset comprising images of road cracks and non-cracked surfaces at disparate scales, to facilitate the dialectical learning of crack characteristics by neural networks.
2. To mitigate the challenges of small crack proportions, sparse feature maps, and complex background interference, we incorporated the CA attention mechanism at the backbone’s terminus of the YOLOv5s model. This mechanism, leveraging unique coordinate encoding, heightens the model’s focus on crack regions, enhancing feature representation and resilience to background noise.
3. To improve detection accuracy for small cracks across different scales, we integrated the Swin Transformer module into the neck of YOLOv5s. This advanced multi-scale self-attention mechanism significantly boosts the model’s ability to capture minute pixel-level details, enhancing crack detection sensitivity and reducing missed and false detections.
4. In view of the possible parameter inflation and model runtime degradation caused by various improvement measures, we optimized the model structure and upgraded the feature extraction backbone to the efficient ShuffleNet V2 architecture. These changes not only stabilize the detection accuracy but also significantly reduce the computational capacity, allowing the model to operate efficiently in low-latency scenarios like drone aerial photography.

## 2. Data and Methods

### 2.1. Data

**Figure 1:**
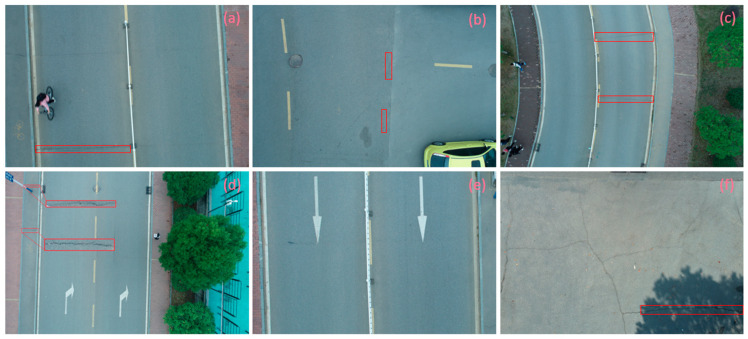
Samples from the UNFSRCI dataset: (**a**) aerial close-up of cracks; (**b**–**d**) close-up, mid-range, and distant small target crack; (**d**) a magnified micro-crack in the shoulder; (**e**) roads without cracks; and (**f**) cracks in the road with tree shadows disturbing.

In terms of data acquisition, we use UAVs to obtain aerial images with high resolution and long range, thereby facilitating a comprehensive understanding of crack distribution over the entire area of interest. Concurrently, the drone maintains a low altitude to capture the intricate details of the crack, thereby fulfilling the requirements of more exacting detection. In order to ensure the diversity and universality of the dataset, we specifically chose a variety of different road types and conditions, photographed under different weather conditions. All images of cracks are meticulously labeled, specifying the location, morphology, and classification of each crack. Furthermore, the images devoid of cracks are subjected to meticulous examination to ascertain the absence of any such defects. Based on this, we constructed a UAV near–far scene road crack images (UNFSRCI) dataset, which provided an adequate quantity of training data for the target detection algorithm. The dataset comprises 1146 images, including 946 images of road cracks and 200 images of crack-free areas. In particular, the crack images comprise 413 long-range images captured by drones and 533 close-up images also taken by drones. In order to further increase the representativeness of the dataset, we purposely took multiple shots under different conditions of light, humidity, and traffic density, and incorporated additional crack-free images, including road deformation fractures and post-pouring strips, into the crack-free sample set. Some samples of the dataset are shown in Figure 1.

**Figure 2:**
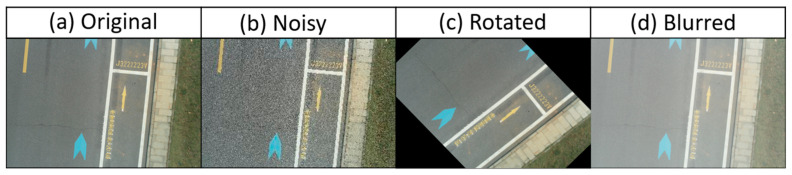
Schematic diagram of data augmentation: (**a**) image without any operation; (**b**) add salt and pepper to the image; (**c**) rotate the image clockwise by 45 degrees; and (**d**) blur the image.

In consideration of the potential disruptions that may arise in the actual situation and to meet the demand for data volume, we employed data augmentation techniques to the collected images to expand the dataset [37], including noise addition, rotation, and blurring, to the collected images, aiming to simulate the complex interference backgrounds likely encountered in practical road crack detection scenarios (as shown in Figure 2). The final dataset comprises 3438 images. Following the methodology proposed by Zhang et al. in 2019 [38], the dataset is divided into three subsets: training, validation, and testing, with a ratio of 6:2:2.

### 2.2. Methods

#### 2.2.1. YOLOv5s

The research in this paper is based on the YOLOv5 model released by Ultralytics in June 2020, which is open-source with more than 250 contributors and is a deep-learning model for use in the field of image processing [39], which excels in accuracy, speed, and deployment flexibility, and is the preferred tool for target detection in real-world applications by developers and researchers [40]. The YOLOv5 model consists mainly of the parts of the backbone, neck, and head, and there are five versions: YOLOv5n (nano), YOLOv5s (small), YOLOv5m (medium), YOLOv5l (large), and YOLOv5x (extra-large).

**Figure 3:**
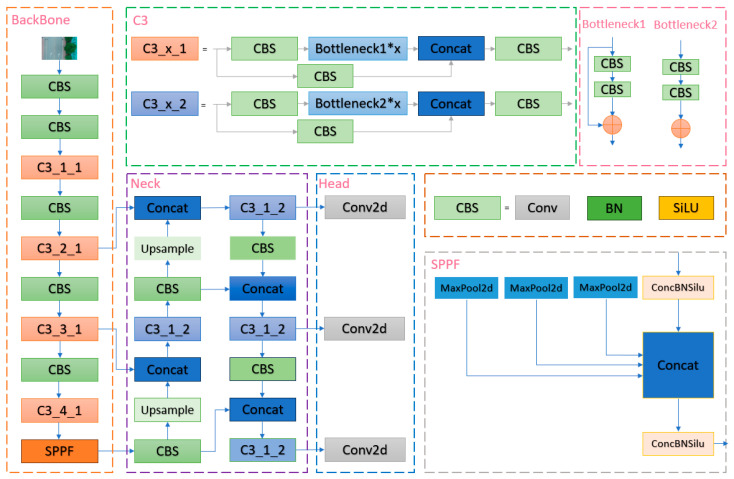
Yolov5s network architecture diagram. The whole network is divided into the backbone, neck, and head. The * sign in the figure indicates that the module can be repeated many times.

The YOLOv5s model is a lightweight version of the YOLOv5 series, with lower computational complexity and faster inference speed, and is more suitable for real-time road crack detection for unmanned aerial systems, which are widely used in this field. In the YOLOv5s model, the k-means method is directly embedded in the overall code, which can adaptively calculate the optimal anchor frame value in different training sets [41]. The backbone primarily consists of CBS [42], CSP Bottleneck with 3 convolutions (C3) [43], and Spatial Pyramid Pooling Fast (SPPF) [44]. CBS is an assembly module that encapsulates convolution, BatchNorm2d, and SiLU activation functions [45]. C3 in the backbone network is an important feature extraction unit designed based on the idea of cross-stage partial network (CSPNet), which can be divided into two forms, C3_x_1 and C3_x_2, according to whether the module has a shortcut [46]; it is mainly used for feature fusion and downsampling, which can simply and efficiently improve the feature extraction capability of the backbone network. The SPPF layer is situated at the end of the backbone and serves to capture multi-scale information of the input image through the utilization of pooling kernels of varying sizes. The dimensionality reduction and compression of the input feature map not only reduce the amount of computation but also improve the receptive field, which is more efficient than its predecessor, Spatial Pyramid Pooling (SPP) [47]. The network neck uses the FPN + PAN structure to merge the features extracted from the backbone with those extracted from the detection layer to obtain richer features [48]. Output three different levels of feature maps and fuse them at the head to achieve multi-scale prediction. A detailed schematic diagram of the YOLOv5s network structure is shown in Figure 3.

#### 2.2.2. ShuffleNet V2

**Figure 4:**
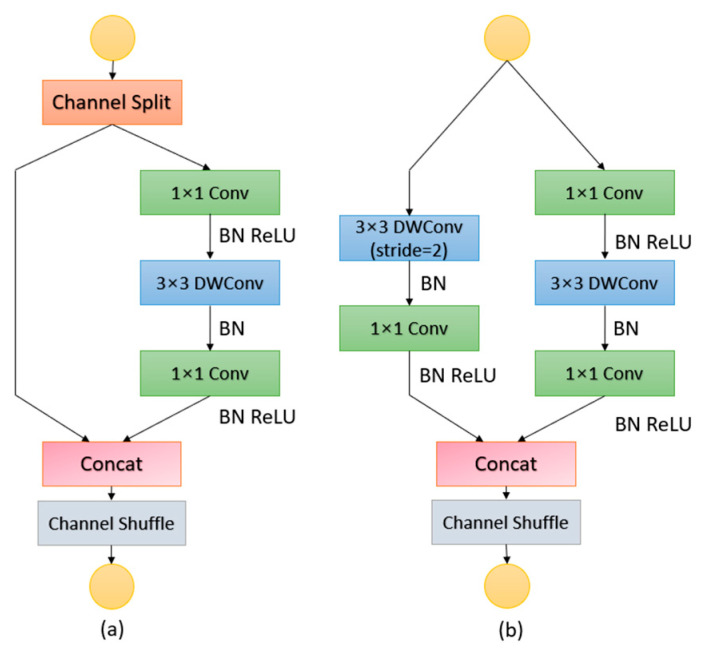
ShuffleNet V2 network architecture diagram. (**a**) our basic unit; and (**b**) our unit for spatial downsampling (2×). DWConv: depthwise convolution.

In the field of image processing, convolutional neural networks (CNNs) have demonstrated efficacy in image detection tasks, as evidenced by recent advancements [49]. However, in parallel with accuracy, computational complexity represents an additional crucial indicator to consider in CNNs; complex network structure tends to increase the amount of computation, resulting in a lower detection speed [50], while specific scenarios like UAV aerial photography have high requirements for low latency. Nowadays, many high-accuracy models have added attention mechanisms that lead to parameter inflation [51], which inflates the amount of data processing, slowing down the operational speed of the model. ShuffleNet V2 is known for its efficient computational efficiency and low number of parameters, which can significantly reduce the complexity of the model and reduce the inference time, and each of its building blocks shows extremely high efficiency, which makes the network structure flexibly carry more feature channels, and then expand the network capacity, while ensuring the computational efficiency, and successfully realizing the model lightweight. To ensure that the computational volume is reduced without decreasing the model recognition accuracy, we introduced ShuffleNet V2 [52]. Aiming at the problem where the feature channels in ShuffleNet V1 are limited by the number of computational resources, ShuffleNet V2 innovatively introduces pointwise group conv and bottle-like structures, along with incorporating the “channel mixing” operation into the network to enhance the information interactivity. The ShuffleNet V2 network mainly consists of two modules: the ShuffleNet V2 basic unit and the ShuffleNet V2 block. The network structure is shown in Figure 4.

As illustrated in Figure 4a, the characteristics of each input unit are partitioned into two branches: one that does not engage in convolution operations and another that performs integrated convolution operations with identical input and output channels. This approach facilitates the balancing of input and output channel sizes while averting network fragmentation. The three successive element-by-element operations, namely, Concat, Channel Shuffle, and Channel Split, have been combined into a single element-by-element operation, thereby reducing the number of element-level operations and significantly increasing the speed of the overall process.

By analyzing the shape of the kernel in a 1 × 1 convolution, it becomes clear that the shape of the convolution kernel typically refers to its spatial dimensions (i.e., height and width) and the number of input and output channels. Consequently, the value of FLOPs for a 1 × 1 convolution can be expressed by Equation (1). The memory access cost (MAC) metric is a measure of the time and resource overhead required to access memory or storage. Its value is calculated using the following equation:

(1) $$B=hwc_{1}c_{2}$$

(2) $$MAC=hw\left(c_{1}+c_{2}\right)+c_{1}c_{2}$$

Here, *B* refers to the FLOPS estimate, h refers to the height of the feature map, w refers to the width of the feature map, $c_{1}$ refers to the number of input channels, $c_{2}$ and refers to the number of output channels.

From Equations (1) and (2), and the mean inequality theorem, we can deduce Equation (3), according to which it can be seen that, when the FLOPS of convolution is constant, when $c_{1}$ and $c_{2}$ are equal, the memory access time and resource consumption are minimized. As a result, the efficiency of network operation can be maximized.

(3) $$MAC\geq 2\sqrt{hwB}+\frac{B}{hw}$$

Given that the splitting operation has already yielded two distinct groups, the two 1 × 1 convolutions are no longer conducted in a group-wise manner, to regulate the number of groups. Following the convolution process, the two branches converge and then undergo a “channel shuffling” operation, which facilitates direct information exchange between the two branches. Subsequently, the subsequent unit commences its operation. Concerning the spatial downsampling unit, the design adheres to the efficient architectural principles of the base unit, as illustrated in Figure 4b.

#### 2.2.3. Coordinate Attention (CA)

**Figure 5:**
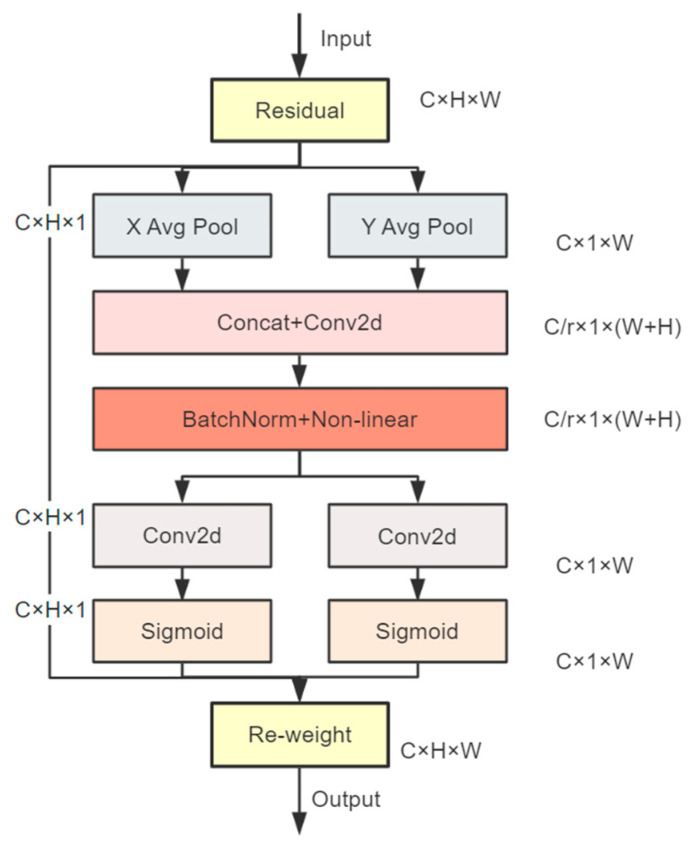
Flow diagram of the CA algorithm.

Attention mechanisms [53,54] are employed to direct models to concentrate on specified elements, thereby facilitating the completion of computer vision tasks. They have gained considerable traction in recent years, particularly within the domain of deep-learning neural networks, where it has consistently demonstrated superior performance in a multitude of complex vision tasks [55,56,57]. In the process of detecting road cracks, the majority of cracks manifest as thin lines in either the transverse or longitudinal directions [58], with the background exhibiting considerable variability. Consequently, the crack constitutes a relatively minor component of the overall detection frame, and the feature mapping tensor of the network output is sparse, rendering it vulnerable to intricate background interference, which reduces detection accuracy. To address this issue, we have incorporated the CA attention mechanism [59] into our approach. The mechanism is an attention mechanism that enhances the feature representation capability by encoding coordinate information. Different from the traditional channel attention mechanism, the CA mechanism not only focuses on the relationship between channels but also introduces location information at the channel level, which enables the model to better focus on the distribution of slender targets (e.g., cracks) in space. Specifically, the CA attention mechanism prioritizes channel-level information, generates weights for each channel by learning the dependencies between channels, and focuses on the narrow region where cracks are located in the spatial dimension through coordinate information. This enhances the characterization of crack features and thus improves the model’s immunity to complex backgrounds. Through this mechanism, the model can identify and locate road cracks more accurately, especially in the presence of complex backgrounds. Figure 5 illustrates the algorithmic process.

It can be seen that CA abandons the traditional global average pooling method and introduces the concept of spatial attention, which encodes one-dimensional (1D) features in both width and height directions. Specifically, given the input X, we use the two spatial extents of the pooled kernel (*H*, 1) or (1, *W*) to encode each channel along horizontal and vertical coordinates, respectively. Therefore, the output of the c-th channel at height h and width w can be expressed by Equations (4) and (5) as follows.

(4) $$z_{c}^{h}\left(h\right)=\frac{1}{W}\sum_{0\leq i<W}x_{c}\left(h,i\right)$$

(5) $$z_{c}^{w}\left(w\right)=\frac{1}{H}\sum_{0\leq j<H}x_{c}\left(j,w\right)$$

The above two transformations aggregate features along two spatial directions to produce a pair of direction-aware feature maps, which make our attention block capture long-range dependencies along one spatial direction and preserve precise positional information along the other spatial direction, which helps the network locate the object of interest more exactly.

**Figure 6:**
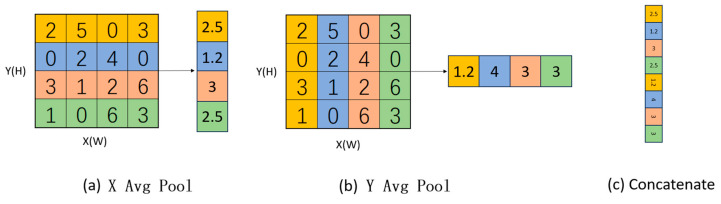
Convolutional pooling process.

After the precise position information is encoded, given the combined feature maps generated by Equations (4) and (5) (the process is shown in Figure 6), we first concatenate them and then apply them to a shared 1 × 1 convolutional transformation function *F*1, leading to the following result.

(6) $$f=\delta \left(F_{1}\left(\left[z^{h},z^{w}\right]\right)\right)$$

where [·,·] denotes the cascading operation along the spatial dimension, δ is the nonlinear activation function, and $f\in {\mathbb{R}}^{C/r\times \left(H+W\right)}$ is an intermediate feature map that encodes spatial information in both the horizontal and vertical directions. r is the reduction ratio of the block size, and then we split f into two separate tensors $f^{h}$ and $f^{w}$ along the spatial dimension. Two additional 1 × 1 convolutional transformations are employed to transform the output tensor into a tensor with the same number of channels as the input X, thereby yielding Equations (7) and (8).

(7) $$g^{h}=\sigma \left(F_{h}\left(f^{h}\right)\right)$$

(8) $$g^{w}=\sigma \left(F_{w}\left(f^{w}\right)\right)$$

Then, the outputs $g^{h}$ and $g^{w}$ were expanded and used as attention weights, respectively. Finally, the coordinate attention block Y for the *c*-th channel is obtained; see Equation (9).

(9) $$y_{c}\left(i,j\right)=x_{c}\left(i,j\right)\times g_{c}^{h}\left(i\right)\times g_{c}^{w}\left(j\right)$$

In conclusion, the coordinate encoding mechanism for input tensors facilitates spatial comprehension by generating two attention maps, one for the horizontal and one for the vertical directions. This approach strikes a balance between capturing long-distance dependencies and preserving positional accuracy, thereby broadening the model’s field of view while maintaining precise localization of objects of interest in complex scenes.

#### 2.2.4. Swin Transformer

**Figure 7:**
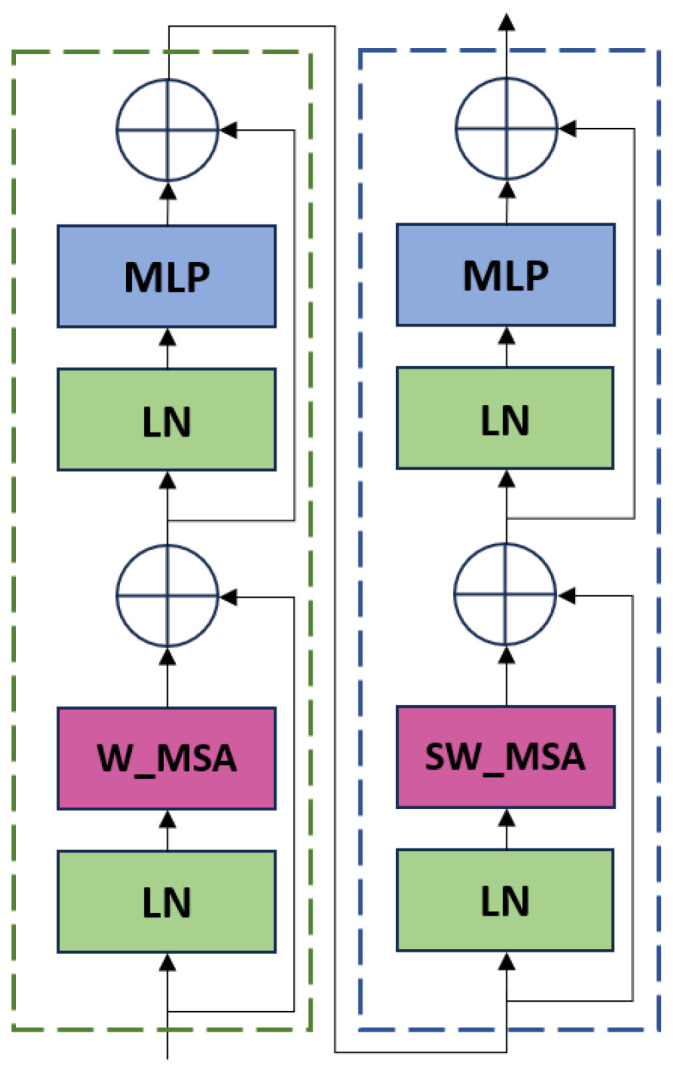
Swin Transformer backbone.

Transformer [60] is an attention-based model that can process dependencies in sequence data without relying on sequence order, as do recurrent neural networks (RNNs). This approach significantly lowers training costs and has made the Transformer the leading architecture in natural language processing (NLP) [61]. Liu et al. [17] introduced a compact version called the Swin Transformer, which uses a hierarchical structure to maintain spatial details often lost in traditional global self-attention methods. To improve the detection of small cracks in images of different sizes, we have incorporated the Swin Transformer into YOLOv5s. This addition allows the model to focus on fine details in images, enhancing its ability to identify tiny cracks. The backbone network configuration is shown in Figure 7.

The Swin Transformer backbone network integrates multi-layer perceptron (MLP), window-based multi-head self-attention (W_MSA), shifted window multi-head self-attention (SW_MSA), and layer normalization (LN). When the input features pass through the Swin Transformer block, it is first accelerated by layer normalization (LN) processing, then screened by window multi-head self-attention (W_MSA), and then weighted by the shift window multi-head attention mechanism to highlight important information and suppress irrelevant information. In this process, residual connections are also used to retain input information, promote gradient transfer, and, ultimately, achieve the purpose of improving the model expression ability and training stability, and the calculated output of these operations is as follows:

(10) $${\overset{\wedge }{z}}^{l}=W-MSA\left(LN\left(z^{l-1}\right)\right)+z^{l-1}$$

(11) $$z^{l}=MLP\left(LN\left({\overset{\wedge }{z}}^{l}\right)\right)+{\overset{\wedge }{z}}^{l}$$

(12) $${\overset{\wedge }{z}}^{l+1}=SW\_MSA\left(LN\left(z^{l}\right)\right)+z^{l}$$

(13) $${\overset{\wedge }{z}}^{l+1}=MLP\left(LN\left({\overset{\wedge }{z}}^{l+1}\right)\right)+{\overset{\wedge }{z}}^{l+1}$$

where ${\overset{\wedge }{z}}^{l}$ and $z^{l}$ denote the output characteristics of the W-MSA module and the MLP module, respectively.

#### 2.2.5. The Improved Road Crack Detection Model

**Figure 8:**
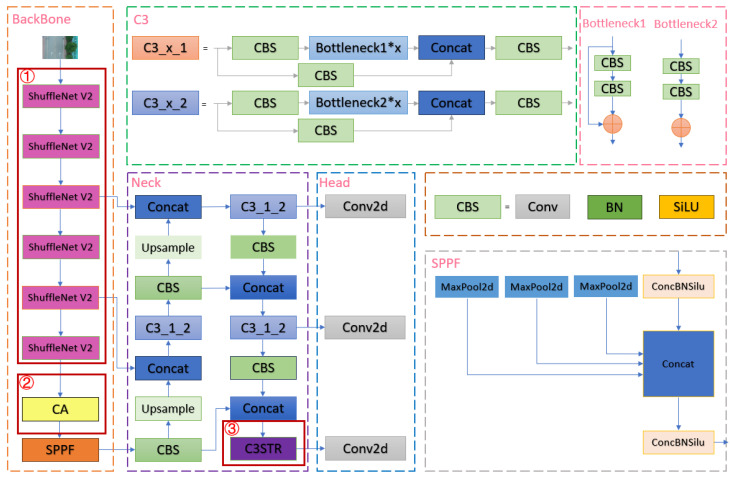
The enhanced model features several key improvements. The * utilized in the illustration denotes that the module can be replicated on numerous occasions.

The primary objective of this study was to modify the trunk and neck of the Yolov5s model. The enhanced model network structure is illustrated in Figure 8.

In Figure 8, the red box highlights several crucial aspects of network improvement: ① The design of the region is to replace all the basic feature extraction units CBS and C3 modules of the whole trunk in the trunk section with ShuffleNet V2 block [62], so as to reduce the amount of computation without decreasing the detection accuracy of the model, and thus to achieve the effect of model lightweight. The ② region is designed by introducing a CA attention mechanism at the end of the trunk to enhance the model’s ability to extract crack features and increase the model’s immunity to complex backgrounds. Area ③ is designed by introducing the Swin Transformer small target detection head in the neck of the model, which can enhance the pixel-level crack feature extraction capability, and then improve the model’s recognition of small target crack features, thus reducing the leakage rate and false detection rate.

These enhancements markedly enhance the model’s performance, not only improving detection accuracy and anti-interference capability in complex backgrounds but also enabling precise identification of crack targets at varying scales. Additionally, the model’s lightweight design renders it adaptable to mobile devices, facilitating real-time, precise, and detailed detection outcomes. This enhanced version of the YOLOv5s model is designated as USSC-YOLO.

## 3. Results

### 3.1. Implementation Details

Different experimental equipment and coding environments may cause significant differences in the experimental results, so we use the same experimental equipment and coding environment throughout the experiment to ensure that the experimental results are scientific and reproducible. The experimental equipment and coding environment are shown in Table 1.

In addition to the experimental instrumentation and coding environment, the hyperparameters of the model can also significantly impact the experimental outcomes. Therefore, before the beginning of the experiment, the hyperparameters of the model were determined by referring to the information [63,64], and the initial training hyperparameter settings are shown in Table 2.

### 3.2. Valuation Metrics

Metrics such as mean average precision (mAP), precision (P), recall (R), and giga floating-point operations per second (GFLOPs) are frequently employed to evaluate the effectiveness of crack detection models [65,66]. In order to assess the efficacy of the training process, this study has elected to utilize the aforementioned four evaluation metrics to ascertain the ultimate performance of the model. Before introducing the aforementioned evaluation indicators, it is pertinent to provide a concise overview of the acronyms that may be utilized throughout the evaluation process, as illustrated in Table 3.

mAP is the arithmetic mean of the average precision (AP) values of all classes, and its manifestations vary according to the different settings of the intersection over union (IoU) threshold, mainly including mAP@0.5 and mAP@ [0.5:0.95]. Specifically, mAP@0.5 is calculated when the threshold of IoU is set to 0.5, meaning a prediction is considered correct only if its IoU with the actual box is 0.5 or higher. This metric evaluates how well the model can locate targets. In contrast, mAP@ [0.5:0.95] is the average of the calculated mAPs for each of the IoU thresholds (from 0.5 to 0.95 in steps of 0.05), providing a broader assessment of model performance under various accuracy conditions and emphasizing both general location accuracy and fine positioning details. The calculation formula is as follows:

(14) $$AP=\int_{0}^{1}P\left(r\right)dr$$

(15) $$mAP50=\sum_{i=1}^{C}\frac{AP_{i}}{C}$$

(16) $$mAP95=\sum_{j=1}^{C}\frac{mAP50_{j}}{C}$$

where *C* refers to the total number of the corresponding samples.

Precision is the ratio of the number of samples with correctly identified cracks to the total number of samples detected as cracks in the model. This metric directly and unambiguously reflects the accuracy and reliability of the model in distinguishing between real and non-fractured specimens. The formula for calculating the accuracy is as follows:

(17) $$P=\frac{TP}{FP+TP}$$

Recall measures how many actual cracks in a dataset are correctly detected and identified by the model. A higher recall indicates that the model misses fewer cracks, showing a better crack detection capability. The recall rate is calculated using the following formula:

(18) $$R=\frac{TP}{TP+FN}$$

GFLOPs, the number of floating-point operations performed a billion times per second, is an important measure of a model’s computational complexity. Typically, the number of model parameters is proportional to the number of floating-point operations required to perform the forward and backpropagation processes. This means that models with more parameters will require more floating-point operations to perform these operations, resulting in a corresponding increase in their GFLOPs values. Conversely, lower GFLOPs values indicate a more compact model structure, suggesting that the model requires fewer computational resources to perform the same task, making it more suitable for resource-constrained environments or application scenarios with high real-time requirements.

### 3.3. Comparing the Effects of Different Lightweight CNNs

In order to meet the strict requirements for low latency in the UAV aerial photography field, we have customized the YOLOv5s model. Specifically, we replaced the CBS and C3 modules, the basic feature extraction units of the model’s backbone network, with more lightweight convolutional neural network components. Among many lightweight convolutional neural networks, we mainly consider MobileNet [67,68] and ShufleNet V1 [69], and ShuffleNet V2. MobileNet dramatically reduces the computational effort and the number of parameters by introducing depth-separable convolution and decomposing the standard convolution into deep convolution and point-by-point convolution, which gives it an excellent performance in embedded devices and real-time applications. However, this simplified operation also leads to a weakened feature extraction capability and low immunity to input perturbations. ShuffleNet V1 overcomes the bottleneck of information flow in grouped convolution by adopting the channel-shuffling technique, which significantly improves the computation efficiency and speed, but its grouping design restricts the transfer of information between channels, which may affect the expression of complex features. ShuffleNet V2, on the other hand, optimizes channel grouping and simplifies computation paths by optimizing channel grouping and simplifying computation paths. ShuffleNet V2, on the other hand, achieves a better balance between speed and accuracy and improves feature extraction efficiency by optimizing channel grouping and simplifying computational paths. In order to further evaluate the performance of these network structures in road crack detection, we conducted comparative experiments, with the experimental results presented in Table 4.

As can be seen from Table 4, in terms of performance indicators such as mAP50, the improvement in the three models is not obvious, but the three sets of new models have a significant impact on the GFLOPs value, especially the combination of YOLOv5s + ShufleNet V2, where the GFLOPs value is reduced by the most, up to 63%, indicating that the introduction of lightweight convolutional neural networks greatly reduces the amount of computation. However, when the complexity of the network structure is not much different, the detection accuracy of ShuffleNet V2 is higher than that of ShuffleNet V1 and MobileNet V2, and is more suitable for the current task.

### 3.4. Effect of Different Attention Mechanisms

In order to enhance the model’s capacity to represent salient features and to increase its resilience to complex backgrounds, we have integrated several mainstream attention mechanisms into YOLOv5s [70,71], including CA, CBAM, SimAM, and SE. The CA mechanism enables the model to accurately focus on the critical regions by capturing directional and positional information; CBAM enhances feature representation through both channel and spatial dimensions to enhance feature representation; SimAM uses statistical information to generate an attention map that effectively highlights important features and suppresses noise; and SE enhances the representation of task-relevant features by learning the dependencies between channels. In order to determine which one is the most suitable for the current detection task, we conducted experiments to explore their impact in enhancing the model’s anti-interference ability and improving the model’s accuracy. The experimental results are shown in Table 5.

mAP is an evaluation method that combines two key indicators: precision and recall. It provides a comprehensive picture of the overall performance of the model in the recognition task. As shown in Table 5, each attention mechanism improves the performance of the model to some extent. However, among the attention mechanisms, SE [72] and CBAM [73] did not significantly improve the mAP value of the model. The CA and SimAM attention mechanisms significantly improved the detection performance of the model, and the variants with the integrated CA increased the values of mAP@50 and mAP@50-95 by 4.4% and 8.4%, respectively, and the variants with the integrated SimAM increased the values of mAP@50 and mAP@50-95 by 3.1% and 7.2%, respectively. It can be seen that the improvement effect brought by the CA attention mechanism is the most obvious. Therefore, we introduced channel attention (CA) in the following improvements. 

### 3.5. Effect of Different Swin Transformer Improvements

The Swin Transformer, as a novel visual transformer, effectively handles the feature extraction problem at different scales through the hierarchical structure and local attention mechanism. Therefore, its introduction into the YOLOv5s model can significantly improve the detection of small targets and complex backgrounds. There are two ways to integrate a Swin Transformer block: one is to replace all the C3 blocks in the model, and the other is to add it to the neck as a stand-alone module. To determine which strategy most significantly improved detection accuracy, we performed separate experiments for each method and recorded the results. The results are displayed in Table 6.

Table 6 shows that Yolov5s + Swin outperforms Yolov5s + Swin_RE_C3 in terms of performance indicators. Although Yolov5s + Swin showed a slight decrease in detection accuracy and recall compared to the benchmark model, the two important metrics of mAP@50 and mAP@50-95 improved by 2.9% and 4.8%, respectively. Therefore, we believe that the integration of Swin Transformer blocks improves the model’s ability to extract crack features from small targets.

### 3.6. Ablation Experiment

Through the previous exploration, we have basically determined the solutions to different problems. Based on this, we further designed ablation experiments using YOLOv5s + ShuffleNet V2 + Swin, YOLOv5s + ShuffleNet V2 + CA, and YOLOv5s + CA + Swin to explore the benefits of a combination of different problem-solving methods. As can be seen from Table 7, the results of the combination of the three modules involved in the improvement are better than the results of changing only one module. It can be seen that there is positive compatibility between the three improvement methods. Therefore, we infer that YOLOv5s + ShuffleNet V2 + Swin + CA is likely to obtain the best performance. However, the possibility that the combination of three modules together will result in a decrease in performance cannot be ruled out. Therefore, we further designed the ablation experiment. The above four schemes were compared with the benchmark module Yolov5s. The results of the experiment are shown in Table 7.

As can be seen from Table 7, the YOLOv5s + ShuffleNet V2 + Swin + CA scheme has high values in all indicators and is the best improved model. Compared with the YOLOv5s benchmark model, the GFLOPs are reduced, the model structure is simpler, and other positive indicators are still improved. Most critically, the mAP@50 improved by 6.3% and the mAP@50-95 by 12%, which means that the crack detection accuracy and robustness of the model have been significantly improved. 

**Figure 9:**
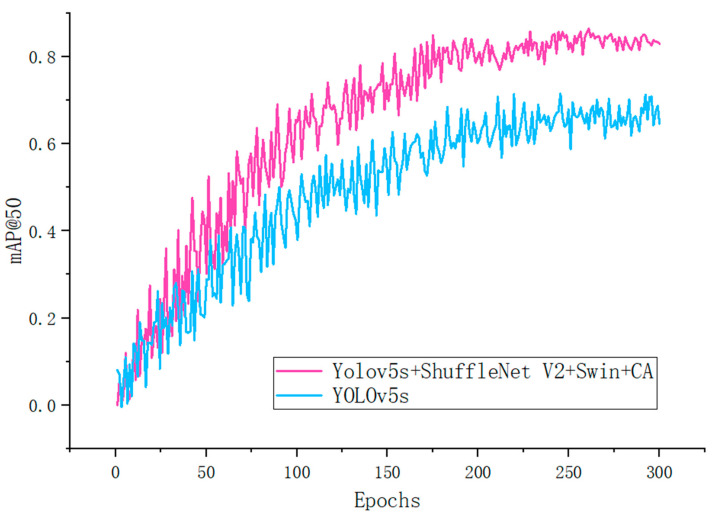
Comparison of ablation experiment trends in mean average precision (mAP@0.5).

In order to visually observe the dynamic changes in the performance of crack detection of the benchmark models YOLOv5s and YOLOv5s + ShuffleNet V2 + Swin + CA, we plotted the relationship between the mAP index and the number of iterations of the two models according to the experimental data (validation data), as illustrated in Figure 9.

As illustrated in Figure 9, the performance trends of the YOLOv5s and YOLOv5s + ShuffleNet V2 + Swin + CA models during road crack detection can be summarized as follows: as the number of iterations increases, the mAP@50 values of both models gradually increase with fluctuations. But, overall, the detection performance of the red line (Yolov5s + ShuffleNet V2 + Swin + CA) is higher than that of the blue line (YOLOv5s). Upon reaching 200 or more iterations, the performance of the red line demonstrates greater stability, with less fluctuation in accuracy. In contrast, the blue line exhibits more variability, suggesting potential instability during the training process. These observations substantiate the superior detection capabilities of the YOLOv5s + ShuffleNet V2 + Swin + CA model in identifying multi-scale cracks within the context of natural landscape disturbances.

### 3.7. Model Performance Comparison Experiments

To further evaluate the performance of the USSC-YOLO model, a comparative experiment was designed. In this paper, our USSC-YOLO model was compared with five of the most popular advanced object detection models (YOLOv5s, YOLOv8s, SSD, YOLOv4 tiny, and Faster R-CNN [74]) to explore the differences in the crack detection effects of different models on the UNFSRCI dataset. The results of the comparison are presented in Table 8.

The results of Table 8 demonstrate that the USSC-YOLO model exhibits superior performance compared to the current mainstream model in terms of mean precision (mAP@50) and mAP@50-95, particularly the value of mAP@50-95. In comparison to YOLOv4, the mAP@50-95 of the USSC-YOLO model has been observed to demonstrate an increase of 15.9%. Furthermore, the GFLOPs of the USSC-YOLO model are 15.6, and the network structure is more concise, which markedly enhances the detection efficiency.

In order to further evaluate the performance of the USSC-YOLO model, we selected three representative images of road cracks (including small cracks and complex backgrounds) taken by UAVs for testing. Subsequently, a comparison was conducted between the test results and those of other models, including Yolov5s, SSD, Fast R-CNN, and others. The results are presented in Table 9 for illustrative purposes.

As can be seen from Table 9, there are significant differences in the detection effects of the four models on road cracks in the actual scenario. This reflects the great difference between the different models in the maintenance efficiency and safety of roads. In Table 9a, the YOLOv5s and SSD models did not accurately identify small target cracks, which implies that, in actual road maintenance, early micro-cracks may be overlooked, thus preventing timely maintenance work. The Faster R-CNN model incorrectly detected the linear watermark on the roadside as cracks, and this misdetection may lead to the unnecessary waste of resources. The USSC-YOLO model, on the other hand, accurately eliminated background interference and accurately detected small cracks, which is important for the timely prevention of road damage and for prolonging the service life of roads. In Table 9b, the YOLOv5s model did not detect small target cracks, which may make the road maintenance personnel miss the best time to repair the cracks, and thus increase the repair cost. Although the SSD model could detect small target cracks, there was a large difference between the marked crack range and the actual crack range, which may lead to inefficiency in repairing the cracks. The Fast R-CNN model can also detect small target cracks; its confidence assessment of these cracks is lower than that of the USSC-YOLO model, which may make the maintenance personnel judge the severity of the cracks insufficiently. In Table 9c, we selected images containing post-pouring tape for testing. The results show that the YOLOv5s, SSD, and Faster R-CNN models incorrectly identify part of the edges of the post-pouring strip as cracks, and such a misdetection may lead to misjudgment and the waste of maintenance resources, even affecting the normal use of the road. Only the USSC-YOLO model is not affected by this interference. Through comparative experiments, the excellent performance of the USSC-YOLO model in multi-scale road crack image detection has been further verified. This not only improves the accuracy of crack detection but also provides reliable data support for road operation and maintenance, which ensures the safety and service life of roads and reduces unnecessary maintenance costs.

## 4. Discussion

As traffic volumes and the accumulation of road travel time increase, the prevalence of road cracks is becoming a significant concern [75,76]. Therefore, it is essential that we detect cracks accurately and quickly. Traditional detection methods are inefficient and costly, and have limitations in real-time detection capability and detection range. With the advancement in scientific and technological knowledge, artificial intelligence has been increasingly applied to the domain of traffic management [77]. Under this background, deep-learning algorithms have become a crucial tool for detecting road conditions, in which single-stage algorithms, although faster in processing speed, are slightly insufficient in detection accuracy. Accordingly, the quest for an algorithm that can guarantee both real-time performance and high accuracy has emerged as a pivotal research topic [78,79,80].

Considering the daily operational status of road traffic flow, and in order not to obstruct traffic, this study employs UAV aerial photography technology to capture detailed images of road traffic conditions daily. In order to accommodate the model’s requirements for comprehensive crack detection and to facilitate the detailed examination of crack characteristics, we captured multi-scale crack-level images encompassing both near and long shots. In light of the lack of a public dataset on crack-free images such as deformation joints and post-pouring strips, we purposely added images containing these strongly intrusive backgrounds to the sample set of crack-free images, thereby constructing a novel UNFSRCI dataset. It is anticipated that this data resource will address some of the deficiencies in the database of drone-photographed crack images, thereby furnishing a more comprehensive array of resources for researchers and practitioners.

In order to ensure that the model can operate efficiently in low-latency requirements scenarios such as UAV aerial photography, this paper selects a lightweight YOLOv5s as the object of improvement. However, the YOLOv5s algorithm exhibits shortcomings in robustness and accuracy, such as missed detection and false detection. Accordingly, we improve the detection performance of YOLOv5s by adding the CA attention mechanism together with the Swin Transformer. However, these two operations lead to a larger number of parameters in the model, thereby increasing the amount of computation, resulting in elevated GPLOPs. This ultimately leads to a decrease in the real-time detection performance of the road crack detection model. In order to address the issue of increased model operation load, we have replaced the entire backbone’s basic feature extraction units of YOLOv5s with ShuffleNet V2. Compared to YOLOv5s, the introduction of ShuffleNet V2 modules results in a significant reduction in GFLOPs, indicating a substantial decrease in computational complexity. In summary, the YOLOv5s + ShuffleNet V2 + Swin + CA variant, obtained through the aforementioned improvements, not only possesses excellent detection performance but is also sufficiently lightweight to be suitable for real-time detection on drones.

This exceptional detection performance brings tangible benefits to road operations. The improved detection accuracy, particularly in identifying small and complex cracks, enables more timely maintenance actions, effectively preventing further road deterioration. This not only reduces long-term maintenance costs but also significantly enhances road safety. As a result, the USSC-YOLO model, when applied in real-world scenarios, not only increases detection efficiency to reduce the burden of manual inspections but also aids road managers in devising more effective maintenance strategies to extend the lifespan of roads and ensure safer transportation.

Ablation experiments conducted on the UNFSRCI dataset confirmed that the USSC-YOLO model achieves a significant improvement in detection performance compared to the original YOLOv5s model. Additionally, when compared to other mainstream models with high detection accuracy, the new model greatly reduces computational load. However, the GFLOPs value of the proposed new model only has a small decrease compared to the original model. Therefore, in the future, we will continue to explore methods for reducing the computational load, aiming to further cause the model to become lightweight without compromising its current excellent detection performance. Currently, the crack images in the UNFRCI dataset come from only one city in China, which limits the diversity of the dataset. Therefore, we plan to collect more road crack images from different regions in the future to further expand the dataset. In addition, the current experimental results are only based on specific hardware conditions. In the future, we will continue to explore the impact of hardware configurations and plan to deploy the model on multiple hardware devices and measure and report the FPS performance under different hardware configurations in detail to determine the optimal application of the model under different hardware conditions.

## 5. Conclusions

Road cracks are the early warning signs of road damage, and their detection is of great importance in ensuring road safety. However, crack detection work with traditional detection methods will inevitably affect the normal operation of traffic. To reconcile this contradiction, this paper relies on visual recognition technology to develop a lightweight model suitable for real-time accurate detection by UAVs.

Specifically, we insert the CA attention mechanism into the backbone network of the YOLOv5s model to improve the model’s ability to extract crack features. Then, the Swin Transformer block is introduced at the end of the neck to reduce the false detection rate. Finally, the basic feature extraction unit of the backbone network is replaced with ShuffleNet V2 to give the model a better performance with fewer parameters.

The experimental results demonstrate that the USSC-YOLO model exhibits a notable enhancement in detection accuracy and a reduction in computational cost compared to YOLOv5s. With its mAP@50 and mAP@50-95, respectively, exhibiting an increase of 6.3% and 12%, the robustness and generalization ability of the model are significantly augmented.

The accurate detection of road cracks is a prerequisite for the intelligent management of road cracks. Currently, we are undertaking a comprehensive analysis of a range of factors, including the coverage area of cracks, covered lanes, and the degree of road use to model the degree of damage caused by cracks. This can assist in the evaluation of the present road safety situation and the repair sequence for crack targets, thereby providing a scientific basis for intelligent management of road cracks.

## Figures and Tables

**Table 1:** Experimental equipment and coding environment.

Train Environmental	Details
Programming language	Python3.9.18
Operating system	Centos7
Deep-learning framework	Pytorch1.12.0 + cu102
Running device	Tesla V100

**Table 2:** Initial training parameters.

Training Parameters	Details
Epochs	300
Batch Size	32
Image Size	640
Learning Rate	0.01
Optimizer	SGD

**Table 3:** Metrics utilized for the evaluation of the models.

Symbols	Meanings
TP	The count of samples where the model accurately detects cracks.
FP	The number of samples where the model incorrectly identifies background as crack.
FN	The count of samples where the model identifies cracks as background.
AP	The area beneath the precision–recall curve is indicative of the average precision in crack detection.
IOU	The overlap rate of the predicted bounding boxes and actual bounding boxes

**Table 4:** Comparative analysis of different lightweight CNNs.

Method	Precision	Recall	mAP50	mAP50-95	GFLOPs
YOLOv5s	0.824	0.639	0.751	0.332	16.0
YOLOv5s + MobileNet	0.854	0.676	0.774	0.336	6.1
YOLOv5s+ ShufleNet V1	0.812	0.668	0.761	0.332	7.9
YOLOv5s+ ShufleNet V2	0.847	0.683	0.776	0.369	5.9

**Table 5:** Comparative analysis of different attention mechanisms.

Method.	Precision	Recall	mAP50	mAP50-95
YOLOv5s	0.824	0.639	0.751	0.332
YOLOv5s + CA	0.824	0.676	0.784	0.360
YOLOv5s + CBAM	0.752	0.618	0.761	0.342
YOLOv5s + SimAM	0.711	0.694	0.774	0.356
YOLOv5s + SE	0.759	0.674	0.771	0.346

**Table 6:** Comparison of different Swin Transformer enhancement methods.

Method	Precision	Recall	mAP50	mAP50-95
YOLOv5s	0.824	0.639	0.751	0.332
YOLOv5s + Swin	0.818	0.634	0.773	0.348
YOLOv5s + Swin_RE_C3	0.795	0.614	0.721	0.326

**Table 7:** Comparison results of ablation experiments.

Method	Precision	Recall	mAP50	mAP50-95	GFLOPs
YOLOv5s	0.824	0.639	0.751	0.332	16.0
YOLOv5s + ShuffleNet V2 + Swin	0.848	0.704	0.779	0.370	12.1
YOLOv5s + ShuffleNet V2 + CA	0.857	0.683	0.786	0.369	12.9
YOLOv5s + CA + Swin	0.838	0.691	0.785	0.358	26.1
YOLOv5s + ShuffleNet V2 + Swin + CA	0.843	0.754	0.798	0.372	15.6

**Table 8:** Comparison of road crack detection effects of various models.

Method	mAP50	mAP50-95	GFLOPs
YOLOv5s	0.751	0.332	16.0
USSC-YOLO	0.798	0.372	15.6
YOLOv4 tiny	0.701	0.313	60.52
YOLOv8s	0.756	0.358	28.8
Faster R-CNN	0.791	0.364	370.21
SSD	0.752	0.338	64.75

**Table 9:** Comparison of visualization experiments.

	Sequences	(a)	(b)	(c)
Method				
Original		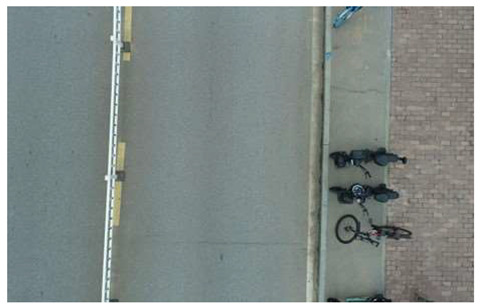	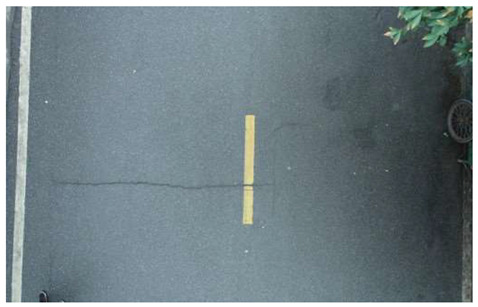	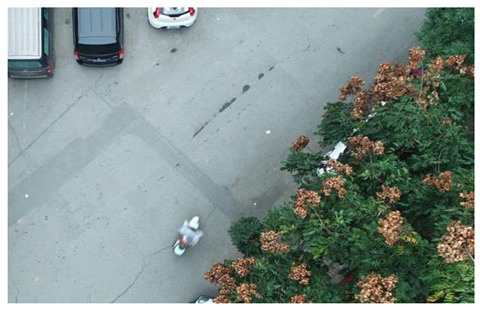
YOLOv5s		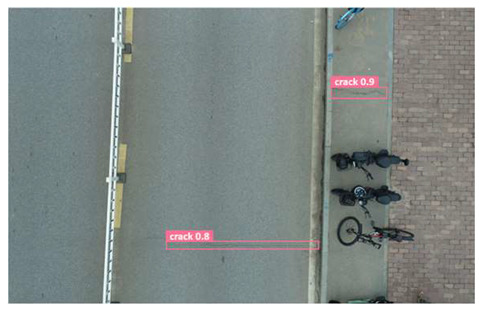	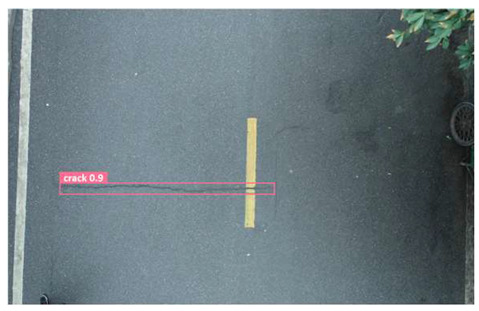	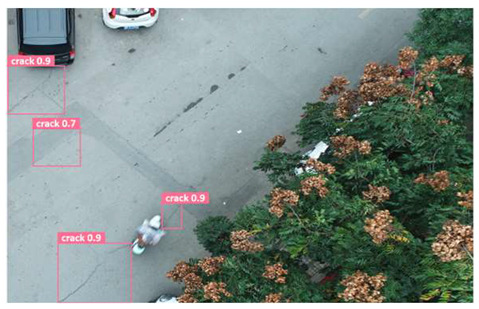
SSD		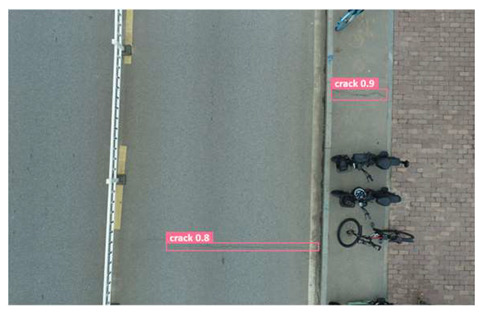	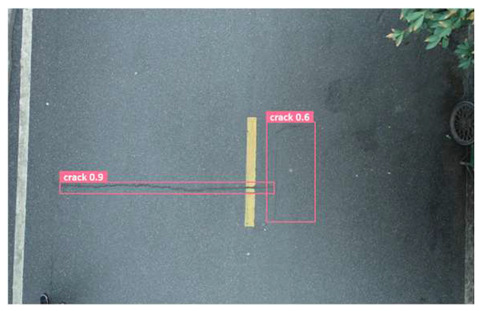	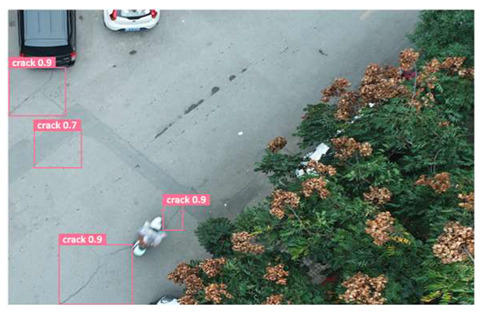
Faster-CNN		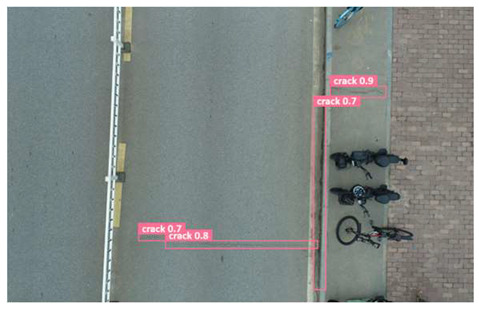	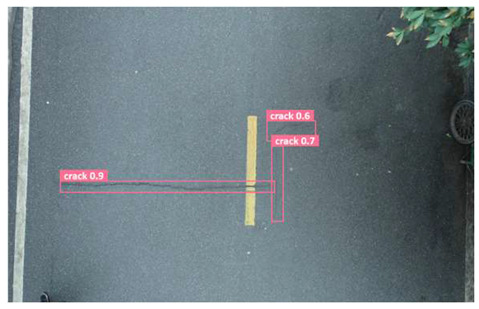	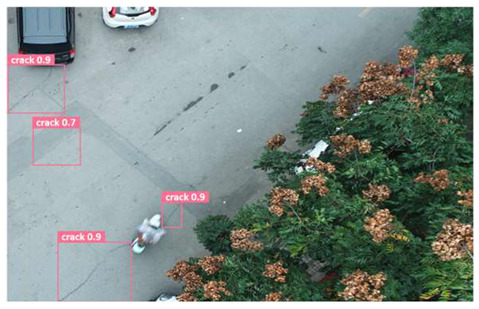
USSC-YOLO		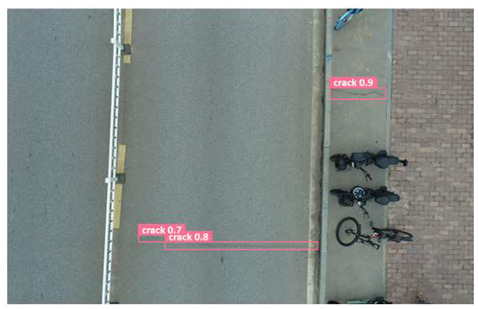	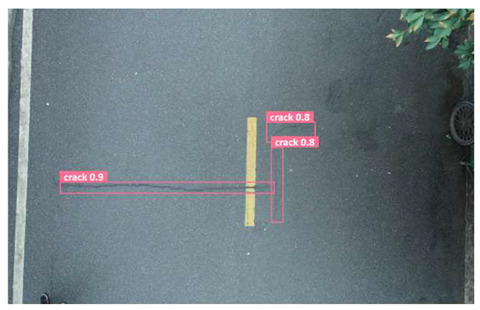	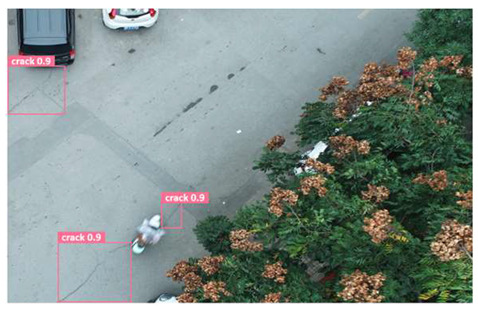

(a) Road crack detection of different sizes under the interference of linear watermarks at the curb. (b) Small targets crack detection with slight interference. (c) Crack detection with background interference similar to cracks.

## Data Availability

The data presented in this study are available upon request from the corresponding author. The dataset and code cannot be shared due to specific reasons.
